# The Trafficking of the Water Channel Aquaporin-2 in Renal Principal Cells—a Potential Target for Pharmacological Intervention in Cardiovascular Diseases

**DOI:** 10.3389/fphar.2016.00023

**Published:** 2016-02-11

**Authors:** Tanja Vukićević, Maike Schulz, Dörte Faust, Enno Klussmann

**Affiliations:** ^1^Max Delbrück Center for Molecular Medicine (MDC) in the Helmholtz AssociationBerlin, Germany; ^2^German Centre for Cardiovascular ResearchBerlin, Germany

**Keywords:** AQP2, AVP, AKAP, PKA, cAMP, NDI, heart failure, SIADH

## Abstract

Arginine-vasopressin (AVP) stimulates the redistribution of water channels, aquaporin-2 (AQP2) from intracellular vesicles into the plasma membrane of renal collecting duct principal cells. By this AVP directs 10% of the water reabsorption from the 170 L of primary urine that the human kidneys produce each day. This review discusses molecular mechanisms underlying the AVP-induced redistribution of AQP2; in particular, it provides an overview over the proteins participating in the control of its localization. Defects preventing the insertion of AQP2 into the plasma membrane cause diabetes insipidus. The disease can be acquired or inherited, and is characterized by polyuria and polydipsia. *Vice versa*, up-regulation of the system causing a predominant localization of AQP2 in the plasma membrane leads to excessive water retention and hyponatremia as in the syndrome of inappropriate antidiuretic hormone secretion (SIADH), late stage heart failure or liver cirrhosis. This article briefly summarizes the currently available pharmacotherapies for the treatment of such water balance disorders, and discusses the value of newly identified mechanisms controlling AQP2 for developing novel pharmacological strategies. Innovative concepts for the therapy of water balance disorders are required as there is a medical need due to the lack of causal treatments.

## Introduction

The kidney has multiple functions including excretion of toxins, control of blood pressure and pH. One of its main functions is water reabsorption from primary urine. The kidney generates more than 170 L of primary urine per day by ultrafiltration of the blood. This huge volume, mainly water, of course needs to be reduced to prevent dehydration. Indeed, only around 1% of the volume is excreted as urine. How does the kidney achieve this?

The functional unit of the kidney is the nephron, of which there are around 1–2 million per kidney. A nephron is a tubular system consisting of several functionally distinct segments. The initial unit is the glomerulus where the blood is filtered at a rate of ~120 ml/min into the surrounding Bowman's capsule. The filtrate flows from there through the proximal convoluted tubule, the descending and ascending limb of Henle, the distal convoluted tubule, and the collecting duct into the bladder. During the passage the volume of the filtrate is reduced and its composition altered. As the final urine, the filtrate reaches the bladder where it is stored until it is cleared.

## Renal water channels: expression pattern and functions

The concentration of the filtrate is achieved mainly by aquaporins (AQPs) that allow diffusion of water across membranes. The water flow is driven by an osmotic gradient established by sodium across the kidney.

**Figure 1:**
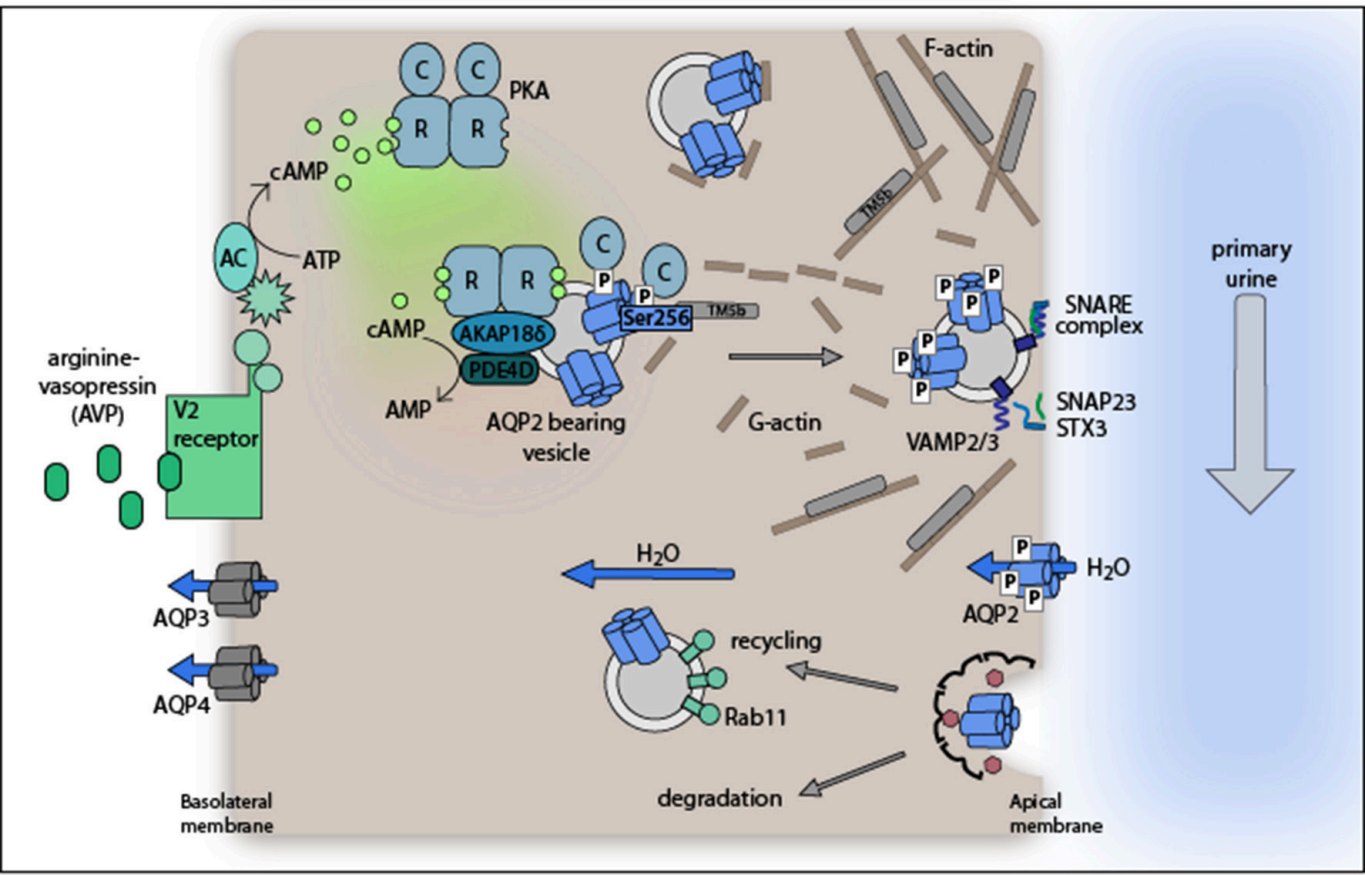
**Model of the arginine-vasopressin (AVP)-stimulated AQP2 translocation from intracellular vesicles into the plasma membrane of renal collecting duct principal cells**. The plasma membrane insertion of AQP2 facilitates water reabsorption from hypoosmotic primary urine. The exocytosis-like process requires PKA phosphorylation of AQP2 at S256. Water exits the cells through water channels AQP3 and 4 constitutively expressed in the basolateral plasma membrane. For details please refer to section Proteins controlling AQP2 trafficking.

There are 13 mammalian AQPs, AQP0-AQP12, that show high structural similarity. They all contain six transmembrane domains, which form a water pore characterized by a dual NPA motif (Preston et al., 1992; Jung et al., 1994; Kosinska Eriksson et al., 2013). All AQPs assemble into homotetramers. Nine AQPs (AQP1-8 and AQP11) are expressed along the nephron with distinct localization to particular segments (Table 1). Crucial roles in water reabsorption from primary urine play AQP1-4. AQP1 in the plasma membrane of epithelial cells facing the lumen of the proximal tubule and descending limb of Henle facilitates reabsorption of 80–90% of water from the filtrate (Nielsen et al., 1993; Schnermann et al., 1998). The ascending limb of Henle is impermeable for water due to the lack of AQPs. From the distal convoluted tubule throughout the collecting duct AQP3 and 4 are constitutively expressed in the basolateral plasma membrane (Ecelbarger et al., 1995; Terris et al., 1995). Thus the apical plasma membrane is water impermeable. However, in the collecting duct the principal cells additionally express AQP2. It resides on intracellular vesicles under resting conditions. Stimulation of vasopressin V2 receptors (V2R) on the basolateral surface of the cells with arginine-vasopressin (AVP) causes a redistribution of AQP2 predominantly into the apical plasma membrane (Figure 1). The insertion facilitates water reabsorption of around 10% of the water from the remaining filtrate (Fushimi et al., 1993; Katsura et al., 1995; Nielsen et al., 2002). In total, the reabsorption of water by AQPs accounts for the reduction of the volume of the initial filtrate from 170 L to around 2–4 L/day of final urine that is on average excreted by an adult.

**Table 1:** **Renal aquaporins**.

**Name**	**Class**	**Exons**	**Synonyms**	**Kidney segment**	**Extrarenal localization**	**Subcellular distribution**	**Function**	**KO mice show**	**References**
AQP1	I	4	AQP-CHIP, CHIP28	PT, DL	Brain, erythrocytes, eye, heart, lung, pancreas, skeletal muscle, vagina	Apical and basolateral PM	Constitutive water reabsorption from pre-urine, tubular cell migration, angiogenesis	Impaired pain sensation, polyuria	Preston and Agre, 1991; Ma et al., 1998; Bai et al., 1999; Pallone et al., 2000; Vacca et al., 2001; Saadoun et al., 2005; Hara-Chikuma and Verkman, 2006; Wang et al., 2008; Kim et al., 2011b; Arrighi and Aralla, 2014
AQP2	I	4	AQP-CD, WCH-CD	CD-PC	Ear, epididymis, vagina	Intracellular vesicles, apical and basolateral PM	AVP-stimulated water reabsorption from urine	Fail to thrive, polyuria	Fushimi et al., 1993; Nielsen and Agre, 1995; Nelson et al., 1998; Merves et al., 2000; Rojek et al., 2006; Kim et al., 2011b; Arrighi and Aralla, 2014
AQP3	II	6	GLIP	CD-PC	Erythrocytes, eye, colon, conjunctiva, lung, skin, vagina	Basolateral PM	Water exit of kidney CD-PC, regulation of epidermal glycerol content	Impaired wound healing, reduced skin hydration, urinary concentration defects	Echevarria et al., 1994; Ishibashi et al., 1994; Ecelbarger et al., 1995; Ma et al., 2000, 2002; Hara et al., 2002; Kwon et al., 2002; Roudier et al., 2002; Kim et al., 2011b
AQP4	I	4	MIWC, WCH4	CD-PC	Brain, eye, lung, muscle, retinal glia, skin, stomach	Basolateral PM	Water exit of kidney CD-PC, regulation of water flow in central nervous system	Impaired vision, hearing, olfaction; urinary concentration defects	Hasegawa et al., 1994; Ma et al., 1994, 1997; Yang et al., 1997; Manley et al., 2000; Li and Verkman, 2001; Zelenina et al., 2002; Hiroaki et al., 2006; Ho et al., 2009
AQP5	I	4	–	CNT, CD-βIC	Ear, eye, lung, salivary glands, placenta, pancreas, vagina	Apical PM	Generation of saliva, tears and pulmonary secretion, unknown renal function	Impaired salivary and sweat secretion, decreased osmotic water permeability across alveolar epithelium	Raina et al., 1995; He et al., 1997; Ishida et al., 1997; Ma et al., 1999; Mhatre et al., 1999; Ma et al., 2000; Krane et al., 2001a,b; Song and Verkman, 2001; Nejsum et al., 2002; Kim et al., 2011b; Procino et al., 2011b; Wu et al., 2013
AQP6	I	4	AQP2L, HKID, KID	CNT, CD-αIC	Brain, vagina	Intracellular vesicles	Urinary acid secretion, nitrate and chloride transport	–	Ma et al., 1996; Yasui et al., 1999a,b; Ohshiro et al., 2001; Ikeda et al., 2002; Liu et al., 2005; Yasui, 2009; Kim et al., 2011b
AQP7	II	6	AQPap	PT	Adipose tissue, brain, heart, intestine, skeletal muscle, testis	Apical PM	Glycerol metabolism, arsenite uptake	Glyceroluria, obesity, smaller islet cells	Ishibashi et al., 1997a, 2000a; Kishida et al., 2000; Nejsum et al., 2000; Liu et al., 2002; Hara-Chikuma et al., 2005; Hibuse et al., 2005; Sohara et al., 2005, 2006a; Laforenza et al., 2013
AQP8	I	6	–	PT	Brain, pancreas, placenta, salivary glands, sperm, testis	Intracellular vesicles, PM	Urea, amonia and ROS transport	Mild hypertriglyceridemia	Ishibashi et al., 1997b; Elkjaer et al., 2001; Yang et al., 2005; Bienert et al., 2007; Saparov et al., 2007; Kobayashi and Yasui, 2010; Tamma et al., 2011b
AQP11	III	3	AQPX1	PT	Brain, intestine, liver, testis, thymus	ER	ER homeostasis, spermiogenesis, salivary gland development	Polycystic kidney disease	Ishibashi et al., 2000b; Morishita et al., 2004, 2005b; Gorelick et al., 2006; Ishibashi, 2006; Yakata et al., 2007; Larsen et al., 2010; Yeung and Cooper, 2010

*AQPap, AQP adipose; AQP2L, AQP2 like; CD-aIC, collecting duct a-intercalated cells; CD-bIC, collecting duct b-intercalated cells; CD-PC, collecting duct principal cells; Class I, classical aquaporins; Class II, aquaglyceroporins; Class III, superaquaporins, unorthodox AQPs, subcellular AQPs; CNT, connecting tubule; DL, descending limb of Henle; ER, endoplasmatic reticulum; GLIP, glucagon-like insulinotropic peptide; HKID, original name of the clone (Ma et al., 1996); KO, knock out; MIP, major intrinsic protein; MIWC, mercurial-insensitive water channel; PM, plasma membrane; PT, proximal tubule; SSC1, small solute channel 1; WCH4, water channel 4*.

This review focuses on mechanisms controlling the localization of AQP2 and the value of such control mechanisms as pharmacological targets in cardiovascular diseases.

## AVP stimulates the redistribution of AQP2 from intracellular vesicles into the plasma membrane

The modulation of the final 10% of water reabsorption in the collecting duct fine-tunes body water homeostasis. Osmoreceptors in the hypothalamus detect changes in blood osmolality that arise, for example, from hypernatremia and hypovolemia, and induce the release of the peptide hormone AVP (antidiuretic hormone) (Verney, 1947; Hayashi et al., 1994). AVP is synthesized in the hypothalamus as a preprohormone. It is released to the pituitary gland and secreted from there to stimulate renal collecting duct principal cells to retain water. As a result, blood volume increases, osmolality decreases, and osmoreceptor signaling declines.

The primary regulator of blood pressure is the renin-angiotensin-aldosterone system (RAAS). Hypotension or hypovolemia induce renin secretion from renal juxtaglomerular cells. Renin converts angiotensin I (Ang I) to angiotensin II (Ang II). AngII acts as a vasoconstrictor, and induces aldosterone and AVP secretion to stimulate renal sodium and water reabsorption. This increases blood volume and blood pressure. As a counterpart natriuretic peptides are secreted by cardiac myocytes upon hypervolemia-induced mechanical stretching of the atrial (atrial natriuretic peptide, ANP) and ventricular (brain natriuretic peptide, BNP) walls. The peptides have vasodilative, natriuretic and diuretic effects to lower blood volume and pressure.

At the molecular level, AVP binds to its cognate G protein-coupled receptor, the V2R, which is located in the basolateral plasma membrane of collecting duct principal cells (Figure 1). The binding of AVP to V2R activates the stimulatory G protein, G_s_. G_s_, in turn activates adenylyl cyclases AC3 and AC6 to convert ATP to cyclic adenosine monophosphate (cAMP). One of the effectors of cAMP is protein kinase A (PKA). It consists of a dimer of regulatory subunits (RIα, RIβ, RIIα, or RIIβ) and two catalytic subunits (Cα, Cβ, Cγ, or PrKX) each bound to one R subunit. Upon binding of cAMP to two sites in the R subunits PKA undergoes a conformational change and the C subunits are released. The free, and now active C subunits phosphorylate nearby targets. PKA phosphorylates serine 256 (S256) in the C terminus of AQP2.

AQP2 undergoes a constitutive recycling: its trafficking from intracellular vesicles into the plasma membrane and endocytic retrieval to its intracellular storage site is in dynamic equilibrium under resting conditions. The equilibrium is shifted toward plasma membrane insertion by the AVP-induced phosphorylation of S256 (Klussmann et al., 2000; Nedvetsky et al., 2009; Moeller et al., 2011). The finding that upon inhibition of endocytosis AQP2 accumulates in the apical plasma membrane of cultured cells regardless of AVP stimulation or S256 phosphorylation emphasizes the importance of the recycling pathway (Lu et al., 2004). It also shows that the redistribution of AQP2 can be induced independently of PKA activation (Nejsum et al., 2005). In addition to the phosphorylation status of S256 that of at least three further C-terminal residues of AQP2 changes in response to AVP stimulation (Fushimi et al., 1997; Katsura et al., 1997; Kamsteeg et al., 2000; Tamma et al., 2010): S261, S264, and S269. S261 is phosphorylated under resting conditions and found intracellularly; AVP triggers its dephosphorylation (Hoffert et al., 2007). In contrast, AVP induces the phosphorylation of S264 and S269 and thereby confers plasma membrane retention properties to AQP2 (Fenton et al., 2008; Hoffert et al., 2008; Moeller et al., 2009).

## Proteins controlling AQP2 trafficking

Several proteins participating in the control of the localization and functioning of AQP2 at various levels have been identified (Table 2). They include, for example, proteins directing exo- and endocytic trafficking, cytoskeletal proteins as the tracks for the trafficking, proteins controlling cAMP signaling such as A-kinase anchoring proteins (AKAPs) and phosphodiestereases (PDEs). A few examples are discussed below.

**Table 2:** **Proteins controlling AQP2 expression and/or localization**.

**Protein**	**Suggested functional implication**	**References**
Actin	Actin-depolymerization promotes AQP2 trafficking to the plasma membrane	Simon et al., 1993; Umenishi et al., 2000; Klussmann et al., 2001; Tamma et al., 2001; Noda et al., 2004b, 2005, 2008
AKAP18δ	AKAP18δ tethers PKA to AQP2-bearing vesicles, most likely facilitating its PKA phosphorylation	Henn et al., 2004
AKAP220	AKAP220 tethers PKA to AQP2-bearing vesicles, most likely facilitating its PKA phosphorylation	Okutsu et al., 2008
Annexin	Annexin II is required for AQP2 trafficking to and/or fusion with the plasma membrane; annexins II and VI belong to a motor complex binding to AQP2; annexins I, II, IV, and V are located on AQP2-bearing vesicles	Barile et al., 2005; Noda et al., 2005; Tamma et al., 2008; Zwang et al., 2009
AP1/2	AP1/2 mediates clathrin-mediated endocytosis of AQP2	Barile et al., 2005; Bouley et al., 2006; Lu et al., 2007
AP-1	AP-1 increases AQP2 transcription	Yasui et al., 1997; Irarrazabal et al., 2008
BIP	BiP selectively binds to phosphorylated AQP2; its functional implication regarding AQP2 is currently unknown	Zwang et al., 2009; Cai et al., 2010
Calcineurin	Calcineurin enhances AQP2 transcription and dephosphorylates AQP2 during GOLGI/vesicle routing, allowing normal trafficking	Valenti et al., 2000; Jo et al., 2001; Gooch, 2006; Li et al., 2007; Rinschen et al., 2011
Calcitonin	Calcitonin induces cAMP-dependent AQP2 trafficking to the plasma membrane	Bouley et al., 2011
Caveolin	Caveolin-1 was suggested to mediate AQP2 internalization	Aoki et al., 2012
CDK	CDK1 and CDK5 were shown to phosphorylate AQP2 at S261	Rinschen et al., 2010
Clathrin	Clathrin forms coated pits for AQP2 endocytosis	Strange et al., 1988; Verkman et al., 1988; Katsura et al., 1995; Sun et al., 2002
COXII	COXII is involved in renal prostanoid synthesis and its inhibition leads to enhanced AQP2 protein abundance	Nørregaard et al., 2005, 2010, 2011; Jensen et al., 2006, 2010; Kim et al., 2008; Kortenoeven et al., 2011
CREB	CREB and CREB-like transcription factors increase AQP2 transcription	Hozawa et al., 1996; Matsumura et al., 1997; Yasui et al., 1997; Umenishi et al., 2006; Yu et al., 2009
CSNK	CSNK phosphorylates S256 during GOLGI transition of AQP2	Brunati et al., 2000; Procino et al., 2003
Dynactin	Dynactin is located on AQP2-bearing vesicles and probably links them to the dynein complex	Marples et al., 1998
Dynamin	Dynamin binds to AQP2 and is involved in the scission of clathrin-coated AQP2-bearing vesicles during endocytosis	Sun et al., 2002; Lu et al., 2004, 2007; Barile et al., 2005; Moeller et al., 2010
Dynein	Dynein mediates the microtubule-associated transport of endocytotic AQP2-bearing vesicles	Marples et al., 1998; Vossenkämper et al., 2007
EPAC	Epac triggers AQP2 translocation to the plasma membrane Ca^2+^-dependently	Umenishi et al., 2006; Yip, 2006; Kortenoeven et al., 2012b
ERK	ERK1/2 increases AQP2 transcription *via* the cAMP/Epac/ERK/CREB pathway; ERK1/2 may mediate S256 phosphorylation under hypertonic conditions; ERK1/2 phosphorylates S261 *in vitro*	Bustamante et al., 2005; Hoffert et al., 2006; Umenishi et al., 2006; Nielsen et al., 2008a; Hasler et al., 2008b; Rinschen et al., 2010
GSK3β	GSK3β enhances PGE2 production by stimulation of COXII, which causes endocytic retrieval of AQP2; GSK3β inhibition was suggested to reduce AVP-induced AC activity	Rao et al., 2005, 2010; Brown et al., 2008; Nielsen et al., 2008b
HSC70, HSP70	Hsc70 and Hsp70 are involved in clathrin-mediated endocytosis of AQP2, were shown to bind AQP2 and suggested to affect AQP2 trafficking to the plasma membrane	Lu et al., 2007; Zwang et al., 2009; Moeller et al., 2010; Rice et al., 2012; Park et al., 2013
Integrin	Integrins α1, α2, α5, and β1 are located on AQP2-bearing vesicles; Integrins α5 and β1 bind to AQP2; Interaction of AQP2 with Integrin β1 promotes renal epithelial cell migration and might regulate AQP2 trafficking *via* cAMP and Ca^2+^	Barile et al., 2005; Wu et al., 2009; Tamma et al., 2011a; Chen et al., 2012
JNK	JNK1/2 may mediate phosphorylation of S261 and S256	Hasler et al., 2008b; Nielsen et al., 2008a; Rinschen et al., 2010
LIP5	LIP5 interacts with AQP2 and facilitates its lysosomal degradation	van Balkom et al., 2009; Boone et al., 2010
MAL	MAL attenuates AQP2 internalization	Kang et al., 2004; Kamsteeg et al., 2007
MLCK	MLCK phosphorylates myosin regulatory light chain (MLC) and facilitates apical sorting of AQP2 by regulating actin filament organization	Chou et al., 2004
Moesin	Moesin was suggested to support the transport of AQP2 to the plasma membrane by modulating actin depolymerization	Tamma, 2005
MUNC18b	Munc18b inhibits fusion of AQP2-bearing vesicle to the plasma membrane by counteracting SNARE complex formation	Procino et al., 2008
Myosin	Myosins and associate proteins were localized on AQP2-bearing vesicles and/or bind to AQP2, Myosin regulatory light chain might facilitate apical sorting of AQP2 by actin reorganization	Chou et al., 2004; Barile et al., 2005; Noda et al., 2005; Nedvetsky et al., 2007
NFκB	NFκB reduces AQP2 gene transcription	Hasler et al., 2008a; Hocherl et al., 2010; Hasler, 2011
p38-MAPK	p38-MAPK phosphorylates AQP2-S261 that is associated with ubiquitination and proteasomal degradation of AQP2	Hoffert et al., 2006; Brown et al., 2008; Hasler et al., 2008b; Nedvetsky et al., 2010; Rinschen et al., 2010
PI3K	PI3K potentiates AVP-mediated increase of AQP2 expression; PI3K mediates endosomal retrieval of AQP2-bearing vesicles	Tajika et al., 2004; Bustamante et al., 2005; Pisitkun et al., 2008
PKA	PKA phosphorylates AQP2-S256 and induces its trafficking to the apical plasma membrane	Kuwahara et al., 1995; Lande et al., 1996; Fushimi et al., 1997; Katsura et al., 1997; Nishimoto et al., 1999; Kamsteeg et al., 2000
PKB	PKB- inhibits GSK3β, which increases the COX-mediated PGE2-production, resulting in reduced AQP2 membrane abundance; PKB inhibits Akt substrate of 160 kDa (AS160), which was suggested to increase plasma membrane abundance of AQP2	Bustamante et al., 2005; Nielsen et al., 2008a; Pisitkun et al., 2008; Jung and Kwon, 2010; Kim et al., 2011a
PKC	PKC induces short-chain ubiquitination of AQP2, leading to its endocytosis and degradation; PKC activation leads to depolymerization of α-tubulin and intracellular localization of AQP2; PKC was suggested to maintain AQP2 transcription by phosphorylation of CREB, PKC is suggested to phosphorylate S256 and S264	van Balkom et al., 2002; Hoffert et al., 2006; Kamsteeg et al., 2006, 2008; Brown et al., 2008; Bagnasco, 2012; Douglass et al., 2012; Thai et al., 2012; Zhao et al., 2012
PKG	PKG was suggested to phosphorylate AQP2-S256, thus increasing its plasma membrane abundance; PKG was suggested to inhibit AVP-dependent AQP2 trafficking by atrial natriuretic peptide (ANP)	Bouley et al., 2000; Brown et al., 2008; Klokkers et al., 2009
PP1/PP2A	PP1 and PP2A inhibition induces AQP2 redistribution to the apical plasma membrane; PP1 binds to AQP2	Valenti et al., 2000; Zwang et al., 2009
RAB	RAB GTPases are located on AQP2-bearing vesicles and regulate its endosomal trafficking	Liebenhoff and Rosenthal, 1995; Barile et al., 2005; Tajika et al., 2005; Nedvetsky et al., 2007; Vossenkämper et al., 2007; Procino et al., 2011a
RAN	RAN binds to AQP2 but its significance regarding AQP2 control is not known	Zwang et al., 2009
RhoA	RhoA stimulates actin-polymerization, which inhibits AQP2 trafficking to the plasma membrane	Klussmann et al., 2001; Tamma et al., 2001; Tamma, 2003
SNAP	SNAP23 and SNAP25 are located on AQP2-bearing vesicles and participate in SNARE complex formation during vesicle and plasma membrane fusion	Inoue et al., 1998; Shukla et al., 2001
SPA-1	SPA-1 binds to AQP2 and stimulates AQP2 trafficking to the apical plasma membrane	Noda et al., 2004a
Synaptotagmin	Synaptotagmin-13 is located on AQP2-bearing vesicles and might be involved in SNARE complex formation during vesicle and plasma membrane formation	Kishore et al., 1998; Barile et al., 2005
Syntaxin	Syntaxins are involved in SNARE complex formation during fusion of AQP2 vesicle and plasma membrane; syntaxins 1A, 2, 3 and 4 are located in the plasma membrane of kidney epithelial cells, syntaxins 5A, 7, 12, 13 and 16 are located on AQP2-bearing vesicles	Mandon et al., 1996, 1997; Gouraud, 2002; Brooks et al., 2003; Barile et al., 2005; Procino et al., 2008; Mistry et al., 2009
TM5b	α-TM5b binds to AQP2, which results in F-actin destabilization and facilitates apical sorting of AQP2	Barile et al., 2005; Noda et al., 2005, 2008; Li et al., 2009b; Park et al., 2013
TONEBP	TonEBP increases AQP2 transcription during hypertonic stress response	Storm et al., 2003; Lam et al., 2004; López-Rodríguez et al., 2004; Hasler et al., 2006; Li et al., 2007; Hasler, 2011
TRPC3	TRPC3 interacts and translocates with AQP2 upon AVP stimulation, its functional implication is presently unknown	Goel et al., 2007, 2010
TRPV4	TRPV4 interacts with AQP2, the functional implication is presently unknown	Galizia et al., 2012
Tubulin	α- and β-tubulin are located on AQP2-bearing vesicles; tubulin forms microtubules, which participate in AVP-elicited apical sorting of AQP2-bearing vesicles and perinuclear positioning of AQP2 after endocytosis	Sabolic et al., 1995; Breton and Brown, 1998; Marples et al., 1998; Shaw and Marples, 2002; Kang et al., 2004; Barile et al., 2005; Tajika et al., 2005; Vossenkämper et al., 2007; Zhao et al., 2012; Yui et al., 2013
Ubiquitin	Ubiquitination at AQP2-K270 mediates AQP2 endocytosis and regulates its proteasomal degradation	Barile et al., 2005; Kamsteeg et al., 2006; Lee and Kwon, 2009; Boone et al., 2011; Lee et al., 2011
VACM-1	VACM-1 targets E3 ligase formation and decreases AQP2 protein abundance	Lee et al., 2011; Le et al., 2012
VAMP	VAMP2 and 3 are located both on AQP2-bearing vesicles and in the plasma membrane and are involved in SNARE complex formation during vesicle and plasma membrane fusion; VAMP8 was suggested to be located on AQP2-bearing vesicles and to be implicated in SNARE complex formation	Franki et al., 1995; Jo et al., 1995; Liebenhoff and Rosenthal, 1995; Marples et al., 1995; Nielsen et al., 1995; Inoue et al., 1998; Gouraud, 2002; Barile et al., 2005; Procino et al., 2008; Wang et al., 2010

*Several proteins were shown to regulate AQP2 expression, abundance, subcellular localization, and degradation. Listed proteins act downstream of receptor activation. For most of them indirect evidence supports their role in AQP2-mediated water reabsorption. AKAP, A-kinase anchor protein, AKAP18δ/AKAP7δ, AKAP220/AKAP11; AP1/2, adaptor protein; AP-1, activator protein; BIP/GRP78/HSP50-5/HSPA5/HSP70-5, Heat shock 70 kDa protein 5/Immunoglobulin heavy chain-binding protein/78 kDa glucose-regulated protein precursor; Calcineurin, Protein phosphatase 2B, PP2B; CSNK, Golgi casein kinase, casein kinase; CDK, Cyclin-dependent kinase; COX, Cyclooxygenase-2; CREB, Cyclic AMP responsive element binding protein; EPAC, Exchange protein activated by cAMP; ERK, Extracellular signal-regulated kinase,ERK1/MAPK3, ERK2/MAPK1; GSK3B, Glycogen synthase kinase 3β; HSC, Heat shock cognate; HSP, Heat shock protein; JNK, c-Jun NH2-terminal kinase; LIP5, Lysosomal trafficking regulator interacting protein-5; MAL, Myelin and lymphozyte associated protein, JNK1/MAPK8, JNK2/MAPK9; MAPK, mitogen activated protein kinase; MLCK, Myosin light chain kinase; Moesin, part of ERM (ezrin/radixin/moesin) protein compex; Munc18b, Unc18-2, Syntaxin-binding protein 2; NFκB, Nuclear factor “kappa-light-chain-enhancer” of activated B-cells; P38-MAPK, p38 mitogen activated protein kinase, MAPK14; PI3K, Phosphoinositide-3-kinase, MAPK14; PKA/B/C/G, Protein kinase A/B/C/G; PP1, Serine/threonine-proteine phosphatase 1; PKB/AKT; RAB, Ras-related protein; RAN, Ras-related nuclear protein; RHOA, Ras homolog family member A; SNAP, Synaptosomal-associated protein 25; SPA-1, Signal-induced proliferation-associated protein 1; TM5b, α-Tropomyosin 5b; TONEBP, tonicity-responsive enhancer binding protein/NFAT5, Nuclear factor of activated T-cells 5/OREBP, Osmotic respone element binding protein; TRPC3, Transient receptor potential cation channel subfamily C member 3; TRPV4, Transient receptor potential cation channel subfamily V member 4; VACM, Vasopressin-activated calcium mobilizing, Cullin 5; VAMP2, Vesicle associated membrane protein/Synaptobrevin; VAMP3, Vesicle associated membrane protein/Cellubrevin*.

### AKAPs

AKAPs are scaffolding proteins. They tether PKA in close proximity of its substrates (Skroblin et al., 2010; Langeberg and Scott, 2015). Global inhibition of AKAP-PKA interactions in a cell culture model of renal principal cells, primary inner medullary collecting duct (IMCD) cells, revealed that AKAP-PKA interactions are crucial for the AVP-stimulated translocation of AQP2 (Klussmann et al., 1999; Klussmann and Rosenthal, 2001). AKAP18δ and AKAP220 are located on AQP2-bearing vesicles to tether PKA in close proximity of AQP2. They apparently facilitate the phosphorylation of S256 (Henn et al., 2004; McSorley et al., 2006; Okutsu et al., 2008).

### PDEs

Termination of cAMP signaling is achieved through hydrolysis of cAMP by PDEs. There are 11 families of PDEs hydrolyzing cAMP, cGMP, or both. PDE4 family isozymes are cAMP-specific, constitutively active and shape local cAMP gradients, thus regulating its availability for PKA activation (Houslay, 2010; Klussmann, 2015; Mika and Conti, 2015). PDE4D directly interacts with AKAP18δ on AQP2-bearing vesicles (Stefan et al., 2007). The presence of PDE4D on AQP2-bearing vesicles prevents an inappropriate plasma membrane insertion of AQP2 and thus water reabsorption in resting IMCD cells. Specific PDE4 inhibition with rolipram did not induce, but enhance the cAMP-stimulated AQP2 translocation into the plasma membrane (Stefan et al., 2007; Szaszák et al., 2008). On the other hand, hyperactive PDE4 diminishes intracellular cAMP and causes AVP resistance in a mouse model (Valtin et al., 1990; Takeda et al., 1991).

### Cytoskeletal components

AQP2 is folded and assembled into homotetramers in the endoplasmic reticulum (ER), passes through the Golgi and is then stored in intracellular vesicles in the perinuclear region. Under resting conditions, AQP2 binds monomeric G-actin (Noda et al., 2004b, 2008). PKA-mediated AQP2 phosphorylation of S256 weakens the interaction. This increases the affinity of AQP2 to the actin-stabilizing protein tropomyosin-5b, withdrawing it from F-actin. Consequently, actin depolymerizes, enabling the vesicle redistribution to the plasma membrane (Noda et al., 2008). AVP-stimulated AQP2 vesicle trafficking to the plasma membrane depends on F-actin depolymerization (Simon et al., 1993). Activation of the small GTPase RhoA induces F-actin-containing stress fibers and tonically inhibits AQP2 trafficking (Klussmann et al., 2001; Tamma et al., 2001). Activated PKA phosphorylates and thereby inhibits RhoA (Lang et al., 1996; Dong et al., 1998), contributing to the AVP-induced destabilization of the actin network. A role of actin in the redistribution of AQP2 is supported by observations in cultured renal collecting duct cells (CD8 cells) treated with the serine/threonine phosphatase 1 and 2a inhibitor, okadaic acid. The agent increased the AQP2 phosphorylation of AQP2 at S256 by around 60% and led to a depolymerization of the actin network. These findings also supported the notion that the redistribution of AQP2 is not exclusively controlled by PKA (Valenti et al., 2000).

### SNAREs

The plasma membrane insertion of AQP2 occurs in an exocytosis-like manner involving the SNARE (soluble N-ethylmaleimide sensitive factor attachment protein receptor) machinery (Liebenhoff and Rosenthal, 1995; Gouraud, 2002). The vesicle-associated membrane proteins (VAMP) 2 and 3 reside on AQP2-bearing vesicles. They interact with apical plasma membrane-located syntaxin 3 and the synaptosome-associated protein (SNAP) 23 to achieve vesicle fusion and the insertion of AQP2 into plasma membrane (Barile et al., 2005; Procino et al., 2008).

### Proteins for AQP2 endocytosis and recycling

Adjustment of body water balance by AQP2-mediated water reabsorption leads to a decline of AVP, followed by internalization of AQP2. AQP2 retrieval from the plasma membrane proceeds *via* clathrin-mediated endocytosis (Sun et al., 2002). AVP stimulates co-accumulation of Hsc70 and AQP2 in the apical plasma membrane (rat kidney sections), and functional Hsc70 is required for AQP2 endocytosis in LLC-PK_1_ cells stably expressing AQP2. AQP2 directly interacts with the endocytotic proteins clathrin, dynamin, and the adaptor protein AP2 (Lu et al., 2007). The phosphomimic mutants S256D and S269D of AQP2 show reduced interactions with the endocytotic machinery, which reduces internalization and increases half-life due to decreased proteasomal degradation (Moeller et al., 2010).

Myelin and lymphocyte-associated protein (MAL), a protein found in glycosphingolipid-enriched membranes and potentially involved in the apical transport machinery forms a complex with AQP2 in LLC-PK_1_ cells (Frank et al., 1998; Martín-Belmonte et al., 2000; Kamsteeg et al., 2007). As phosphorylation of S256 strengthens the interaction with MAL and MAL attenuates AQP2 internalization, this is in line with the above-described reduced endocytosis to explain the stabilization and accumulation of pS256 in the apical plasma membrane.

From the plasma membrane AQP2 is recycled or directed for degradation. The motor protein myosin Vb, its receptor on AQP2-bearing vesicles, Rab11, and the adaptor protein Rab11-FIP2 are essential for recycling through the Rab11-dependent recycling pathway (Nedvetsky et al., 2007). Positioning of AQP2-bearing vesicles in their perinuclear storage region involves microtubules (Vossenkämper et al., 2007).

### Ubiquitin

Under resting conditions AQP2 is phosphorylated at S261 by, amongst others p38 MAP kinase (Nedvetsky et al., 2010). The phosphorylation is a signal for AQP2's mono- and poly-ubiquitination. The short-chain ubiquitination of AQP2 at K270 increases its endocytosis under resting conditions (Kamsteeg et al., 2006). In primary IMCD cells, AVP causes a decrease in the phosphorylation of S261 within 30 min of exposure. This reduces polyubiquitination and prevents AQP2's proteasomal degradation resulting in an increase in its abundance. Thus, AQP2 expression adapts quickly to water availability: thirsting increases, whereas water overload reduces AQP2 levels (Nedvetsky et al., 2010). The AVP-induced reduction of polyubiquitination of AQP2 implies that its recycling *via* the Rab11 pathway requires its deubiquitination.

AQP2 short-chain ubiquitination and subsequent internalization can occur independently of the AVP-PKA axis upon activation of protein kinase C (PKC) (Han et al., 1994; van Balkom et al., 2002; Kamsteeg et al., 2006). Prostaglandin 2 (PGE_2_) or dopamine counteract AVP and induce AQP2 retrieval from the apical plasma membrane (Zelenina et al., 2000; Edwards and Brooks, 2001). The underlying pathway is not fully elucidated. In addition to PKC it may involve modulation of the ubiquitination of AQP2 (Hébert et al., 1990; Tamma et al., 2003b; Nejsum et al., 2005).

## Dysregulation of AVP-mediated water reabsorption causes water balance disorders

**Figure 2:**
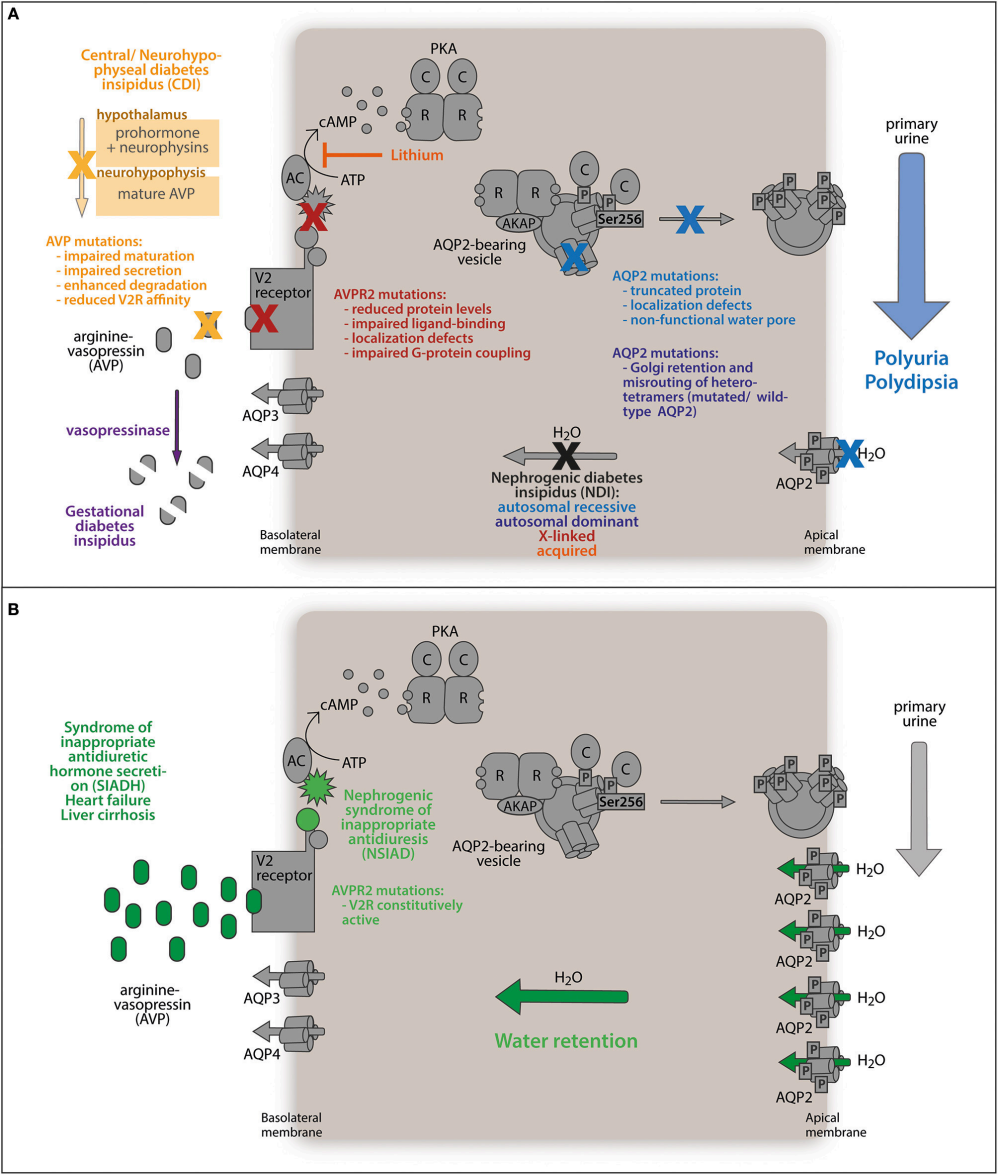
**Water balance disorders**. **(A)** Diabetes insipidus describes the abnormal loss of hypoosmotic urine (polyuria) along with increased thirst (polydipsia). The antidiuretic peptide hormone arginine-vasopressin (AVP) is synthesized in the hypothalamus as a prohormone. Mature AVP is secreted from the neurohypophysis to stimulate the kidneys to retain water. Mutations in the AVP gene impede correct processing or reduce its affinity for the V2 receptor, which precludes AVP-mediated water reabsorption from primary urine and is termed central diabetes insipidus (CDI). In the rare case of gestational diabetes insipidus, placenta-released vasopressinase degrades AVP, counteracting antidiuretic signaling. Similarly, mutations in the gene encoding the V2 receptor (AVPR2) render renal principal cells AVP-resistant. Defects in AQP2 protein expression, channel function, intracellular sorting, or membrane translocation disrupt the responsiveness of principal cells to antidiuretic stimuli and maintains low water permeability of the kidney collecting duct. **(B)** Deregulated accumulation of AVP enhances antidiuretic stimulation of renal principal cells through the V2 receptor and triggers enhanced AQP2-conducted water reabsorption from the primary urine urine (SIADH, heart failure, liver cirrhosis). The same effect is caused by a mutation in the AVPR2 gene constitutively activating the receptor. Both defects abnormally elevate water retention.

Several diseases are associated or caused by dysregulation of AVP-mediated water reabsorption (Figure 2). Elevated levels of AVP as in the syndrome of inappropriate antidiuretic hormone secretion (SIADH), late stage heart failure, and liver cirrhosis cause excessive water retention. Defects preventing the insertion of AQP2 into the plasma membrane lead to diabetes insipidus.

## Decreased AVP-mediated water reabsorption causes diabetes insipidus

Patients suffering from diabetes insipidus display a limited ability to concentrate urine. This leads to polyuria and polydipsia (Knoers, 1993; Bockenhauer and Bichet, 2015), and if untreated to severe dehydration, hypernatremia, and hyperchloremia (Multari et al., 2001; Qureshi et al., 2014).

Diabetes insipidus may be inherited or acquired. In central diabetes insipidus (CDI) mutations in the gene encoding AVP lead to insufficient amounts of AVP released from the pituitary gland. The consequence is a compromised concentrating ability of the collecting duct due to a low level of V2R stimulation and abnormally low AQP2 levels, resulting from the lack of AVP-dependent AQP2 gene expression (Ishikawa, 2000). The prevalence of CDI is 1 in 25,000 (www.orpha.net). An animal model reproducing CDI is the Brattleboro rat. The rats lack AVP and the expression levels of AQP2 are decreased (Sokol and Zimmerman, 1982; Kishore et al., 1996).

Mutations in the V2R gene (X-chromosome, Xq28) cause X-linked NDI, which accounts for ~90% of all congenital NDI forms (Peters et al., 2007; Heinke and Labudde, 2012). Due to the lack of a functional V2R gene, the AQP2 level is decreased and AQP2 does not translocate into the plasma membrane. The prevalence of X-linked NDI is 3.7 in 1 million males (Bichet et al., 1992). Interestingly, in the Canadian province Nova Scotia the incidence is much higher with 58 in 1 million male live births, a founder effect discussed as the “Hopewell hypothesis” (Bichet et al., 1993; Arthus et al., 2000). The X-linked inheritance is obviously more apparent in males due to the lack of a second X chromosome. Even after administration of dDAVP (see below) the male patients do not show signs of improvement (Knoers and Deen, 2001). An animal model reproducing the phenotype is the inducible V2R knockout mouse model (Li et al., 2009a).

The remaining ~10% of the persons with congenital autosomal recessive and autosomal dominant forms of NDI carry mutations in the AQP2 gene (Deen et al., 1994; Park et al., 2014). Autosomal dominant forms were observed in patients with frame shift mutations producing a C-terminally elongated AQP2 protein (721delG/727delG) or a mutation leading to an E258K substitution (Mulders et al., 1998; Kuwahara et al., 2001; Marr et al., 2002b). When overexpressed in *Xenopus* oocytes, the E258K, and AQP2-727delG versions were misrouted to the Golgi and late endosomes, respectively (Mulders et al., 1998; Marr et al., 2002b). The dominant-negative effect seems to be conferred by the formation of heterotetramers with wild type AQP2 that fail to translocate to the plasma membrane in response to AVP (Marr et al., 2002b). Disruption of the PKA consensus sequence by the substitution R254L interferes with the AVP-induced PKA phosphorylation of S256 and also leads to autosomal dominant NDI (de Mattia et al., 2005). The AQP2 version S256L, associated with congenital progressive hydronephrosis, cannot be phosphorylated and causes a severe urine concentration defect in mice, underpinning the importance of S256 for controlling water homeostasis (McDill et al., 2006).

Most of the more than 30 known AQP2 mutations associated with hereditary nephrogenic diabetes insipidus cause autosomal recessive NDI (Fujiwara and Bichet, 2005; Park et al., 2014; Cen et al., 2015). The mutations predominantly cause ER retention and impair protein stability and proper channel function (Deen et al., 1995; Mulders et al., 1997; Goji et al., 1998; Marr et al., 2002a). In comparison to the autosomal dominant mutants, the recessive ones such as AQP2-R187C do not form heterotetramers with wild type AQP2 (Kamsteeg et al., 1999).

There are several animal models available for studying AQP2 in the pathophysiology of NDI (Boone and Deen, 2009). Mice globally lacking AQP2 or mice expressing the T126M mutant die within 2 weeks of age (Yang et al., 2001; Rojek et al., 2006). The collecting duct-specific knockout of AQP2 enabled survival. In addition, mouse models with an amino acid substitution in the PKA phosphorylation site (S256A; McDill et al., 2006), an F204V substitution (Lloyd et al., 2005) or a complete deletion of the C-terminus were established to study recessive NDI (Shi et al., 2007). Sohara et al. presented the first mouse model for autosomal-dominant NDI, in which the assembly of mutant with wild type AQP2 into heterotetramers supported the *in vitro* findings (Sohara et al., 2006b). Using this model the PDE inhibitor rolipram was identified as a potential drug to treat autosomal-dominant NDI.

The most common form of NDI is acquired NDI. One of the main causes is the long-term treatment of bipolar disorders with lithium. This causes acquired NDI in up to 40% of the patients (Sim et al., 2014). In elderly patients a 6-year prevalence rate of 3% was observed (Rej et al., 2014). The uptake route of lithium into collecting duct principal cells is through the epithelial sodium channel (ENaC). Lithium inhibits adenylyl cyclases and thereby cAMP synthesis and, consequently, the cAMP-dependent signaling that controls AQP2 trafficking and expression. A marked decrease in AQP2 expression in human and rat medullary collecting ducts has been reported with long-term lithium carbonate treatment (Blount et al., 2010).

Gestational DI is another form of acquired NDI. It manifests during pregnancy in the third trimester and in the early postpartum period. During pregnancy, the threshold level of serum osmolality to induce AVP secretion is lowered. The metabolic AVP clearance is increased due to increased levels of the trophoblast-produced aminopeptidase vasopressinase, which can lead to a transient form of DI (Barron et al., 1984; Durr et al., 1987; Davison et al., 1989). Gestational DI has a prevalence of 2–4 in 100,000 pregnancies and can be treated with dDAVP (see below), as this AVP analog is resistant to degradation by vasopressinase (Ananthakrishnan, 2009). Placental abruption can cause acute postpartum DI owing to high amounts of placental vasopressinase released into the circulation (Wallia et al., 2013). Occurrence of gestational DI is elevated in women with preeclampsia, HELLP syndrome (Hemolysis, Elevated Liver enzymes, and Low Platelets) or liver steatosis. These conditions present defective hepatic vasopressinase degradation (Katz and Bowes, 1987; Usta et al., 1994; Ellidokuz et al., 2006).

Bilateral ureteral obstruction (BUO) can cause acquired NDI with a marked decrease in AQP2 abundance (Jensen et al., 2009; Radin et al., 2012). In a rat model, the AQP2 level drops to a fourth of the control within 24 h upon induction of BUO and the phosphorylation of S256 is decreased (Stødkilde et al., 2011). Even 1 week after the release of BUO and when the urine output had become normal, AQP2 levels reached only half those of the controls. Upon dehydration, the affected rats were not able to efficiently concentrate urine (Frøkiaer et al., 1996), indicating that reduced levels of AQP2 are the main limiting factor during recovery from polyuria caused by BUO.

Acquired NDI can also be caused by hypercalcemia (Khairallah et al., 2007; Kamath et al., 2013), hypokalemia (Celik et al., 2014), and secondary hyperaldosteronism (Lee et al., 2010; Qureshi et al., 2014).

## Increased AVP-mediated water reabsorption causes water retention and hyponatremia disorders

In SIADH, late stage heart failure, and liver cirrhosis elevated levels of AVP lead to diluted plasma with hyponatremia with or without hypervolemia. Hyponatremia is a physiological response to maintain an adequate circulating blood volume but turns into an electrolyte disorder when serum sodium drops to less than 135 mEq/L.

SIADH is the most frequent cause of hyponatremia, accounting for approximately one-third of all cases (Ishikawa, 2015). The abundance and plasma membrane localization of AQP2 is increased (Kortenoeven et al., 2013), which leads to excessive water retention and hypervolemic (dilutional) hyponatremia (Fenske and Allolio, 2010; Braun et al., 2015). A mutation in the V2R gene that causes nephrogenic syndrome of inappropriate antidiuresis (NSIAD) leads to constitutively active receptors triggering SIADH-like symptoms without elevated AVP levels (Feldman et al., 2005).

In humans with heart failure and in several animal models of heart failure, RAAS system activity, the non-osmotic release of AVP and the expression level and membrane localization of AQP2 are significantly increased. The consequence is water retention with increased circulatory blood volume (Radin et al., 2012; Cui et al., 2015; Ishikawa, 2015). An excessive circulatory volume results in hypervolemic hyponatremia. These patients have a poor prognosis (Noveanu et al., 2011). However, recent studies of heart failure in rat models revealed increased AQP2 protein abundance even in the presence of normal AVP and unchanged plasma sodium levels (Hadrup et al., 2004). Thus in heart failure AQP2 may be up-regulated more generally than previously thought (Brønd et al., 2013).

Liver cirrhosis is associated with a non-osmotic AVP release, water retention and hyponatremia (John and Thuluvath, 2015). The NO concentration in the plasma of cirrhosis patients correlates with the extent to which AQP2 levels are increased (Chung et al., 2010). However, the underlying mechanisms modulating water reabsorption through AQP2, and sodium excretion are unknown (Wilson et al., 2013).

Nephrotic syndrome is similar to heart failure and liver cirrhosis with regard to increased AVP levels and water retention (Apostol et al., 1997; Bou Matar et al., 2012). In contrast to heart failure and liver cirrhosis the nephrotic syndrome shows an “escape” from AVP in the renal collecting ducts. This “escape phenomena” is characterized by a notable down-regulation of AQP2 expression despite high AVP levels. Water balance disorders associated with “escape” phenomena are characterized by an ability to prevent water retention and hyponatremia *via* secondary natriuresis (Brønd et al., 2004; Verbalis, 2006). A possible mechanism involved in the AVP “escape” includes reduced V2 receptor binding and uncoupling of the V2 receptor from its cognate G protein resulting in abnormally low cytosolic cAMP concentrations upon dDAVP stimulation (Ecelbarger et al., 1998; Tian et al., 2000; Jonassen et al., 2003; Brønd et al., 2004).

## Established pharmacological treatments of diabetes insipidus and new options

### Desmopressin (dDAVP)

CDI is commonly treated by the administration of an AVP analog, desmopressin (dDAVP), for example, *via* a nasal spray or infusion (van Balkom et al., 2004; Kortenoeven and Fenton, 2014). Chronic treatment with dDAVP increases the AQP2 level in apical plasma membranes of both cortical and inner medullary collecting duct principal cells, and decreases urine output usually drastically (Agarwal and Gupta, 2008; Kortenoeven and Fenton, 2014).

### Diuretics

The main strategy for the treatment of X-linked NDI is to bypass non-functional V2R and restore physiological expression levels of AQP2 as well as AQP2 trafficking. Thiazides are widely used in patients with NDI. They decrease urine volume, but the mechanism by which they exhibit their paradoxical antidiuretic effect is poorly understood (Kim et al., 2004a). It was proposed that thiazides inhibit reabsorption of sodium and chloride in the distal collecting duct and thus increase the osmolality of the urine (Bockenhauer and Bichet, 2014).

Hydrochlorothiazide (HCTZ) combined with amiloride is the main therapy in patients with lithium-induced NDI (Sinke et al., 2014). Treatment with thiazides can decrease the glomerular filtration rate, which causes a reduced excretion of lithium, resulting in lithium intoxication. Amiloride, a potassium sparing diuretic, interferes with lithium uptake by blocking ENaC, and for this reason is combined with thiazides (Bedford et al., 2008; Kishore et al., 2015). Also a combination of HCTZ with the NSAID, indomethacin (see below) reduces urine output more effectively than the administration of thiazides alone (Kortenoeven and Fenton, 2014). The expression of AQP2 was substantially reduced in the collecting ducts of lithium-treated rats and was increased in the presence of amiloride (Bedford et al., 2008).

### Statins

Statins are lipid-lowering drugs used to reduce cardiovascular risks. They competitively inhibit hepatic 3-hydroxy-3-methylglutaryl-coenzyme A reductase catalyzing the conversion of HMG-CoA to mevalonate. Thereby statins inhibit the production of isoprenoid intermediates such as farnesyl pyrophosphate (FPP), geranylgeranyl pyrophosphate (GGPP). A range of proteins undergoes post-translational modifications by addition of isoprenoid pyrophosphates (Verhulst et al., 2004). FPP and GGPP act as lipid anchors required for membrane tethering and activation of heterotrimeric G proteins and small GTPases, amongst them RhoA (Li et al., 2011; Szygula-Jurkiewicz et al., 2014). Statins inhibit RhoA isoprenylation, leading to the accumulation of inactive RhoA in the cytoplasm (Liao and Laufs, 2005).

In MCD4 cells, mouse collecting duct cells stably expressing AQP2, statins inhibit the isoprenylation and thus activation of RhoA (Procino et al., 2010). This causes F-actin depolymerization and an AVP-independent increase in AQP2 plasma membrane localization (Bouley et al., 2008; Procino et al., 2010, 2011a; Li et al., 2011; Wade, 2011). Statins also activate the NO/cGMP pathway (Tamma et al., 2003a; Rikitake and Liao, 2005; Maher et al., 2009) and thus may induce AQP2 phosphorylation at S256 and thereby induce the insertion into the plasma membrane. Incubation of LLC-PK_1_ and MCD4 cells with simvastatin resulted in AQP2 plasma membrane accumulation in a dose-dependent manner due to reduced clathrin-mediated endocytosis (Procino et al., 2012). Short-term exposure to simvastatin produced no change in cholesterol plasma membrane levels, but increased AQP2 accumulation in the apical membrane of principal cells in Brattleboro rats. This increased water reabsorption and urine concentration (Li et al., 2011; Bonfrate et al., 2015). In adult Munich-Wistar male rats subjected to BUO for 2 h atorvastatin improved urinary concentrating ability by reversing ureteral obstruction-induced down-regulation of AQP2 (Danilovic et al., 2012). Fluvastatin increased AQP2 plasma membrane abundance in wild-type mice. In the same study when X-linked NDI mice were treated with secretin plus fluvastatin, urine volume was reduced by nearly 90% and the urine osmolality was doubled. Fluvastatin promoted AQP2 trafficking to the plasma membrane whereas secretin increased intracellular stores of AQP2 (Procino et al., 2014). Additional experiments in mice indicated that fluvastatin increases AQP2 membrane accumulation by altering the prenylation status of key proteins regulating AQP2 trafficking such as RhoA (Procino et al., 2011a).

In a recent study, 24 naïve hypercholesterolemic patients were treated with simvastatin (20mg/day for 12 weeks). This rapidly and significantly enhanced urine excretion of AQP2, and decreased the 24 h diuresis while urine osmolality increased. This occurred similarly and persistently in patients who were treated with statins in the long-term, i.e., for at least 1 year (Procino et al., 2015).

Collectively, the data suggest that statins provide novel opportunities for the treatment of X-linked NDI as their target is independent from V2R.

### Prostaglandins

Prostaglandin E_2_ (PGE_2_) is synthesized and released in the collecting duct (Olesen et al., 2011). It is one of the major cyclooxygenated metabolites of arachidonic acid that stimulates all four G protein-coupled E-prostanoid receptors, EP_1_-EP_4_ (Hao and Breyer, 2008). PGE_2_/EP_1_ signaling elicits a diuretic response *via* the Gα_q_-mediated activation of PKC, triggering AQP2 internalization (Bachteeva et al., 2007). However, an EP_1_-mediated decrease in AQP2-induced water permeability was only observed in frog urinary bladder, but not in collecting ducts (Bachteeva et al., 2007). EP_3_ is coupled to Gα_i_ and reduces cAMP synthesis upon activation (Sugimoto et al., 1992), thus decreasing AQP2 expression and membrane localization (Zelenina et al., 2000). EP_3_-signaling also stimulates RhoA (Tamma et al., 2003b), probably mediated by the Gα_12∕13_-dependent activation of Rho guanine nucleotide exchange factors (GEFs) (Yamaguchi et al., 2000; Wells et al., 2002). GEFs directly activate RhoA and thus attenuate AQP2 membrane trafficking (Klussmann et al., 2001; Tamma et al., 2001). The activation of the different signaling pathways is most likely due to different EP_3_ splice variants (Namba et al., 1993; Hatae et al., 2002). The observation of various cellular responses might also be the result of the differential expression of PGE_2_ receptors in model systems derived from different renal sections of diverse species (Olesen and Fenton, 2013).

EP_2_ and EP_4_ couple to Gα_s_ (Alexander et al., 2011). The stimulation of each receptor results in the increased phosphorylation of AQP2-S264, whose effect is presently unknown (Olesen et al., 2011). Only EP_2_ signaling increases the cytosolic cAMP concentration AVP-independently (Breyer and Breyer, 2001) and stimulates the phosphorylation of AQP2 at S256 and S269 (Olesen et al., 2011). However, there are contradictory data on whether EP_2_ is expressed in the collecting duct at all (Sugimoto et al., 1994; Breyer et al., 1996; Morath et al., 1999; Jensen et al., 2001; Regan, 2003; Hao and Breyer, 2008). EP_4_ is widely spread in the collecting duct (Sugimoto and Narumiya, 2007). Its activation enhances AQP2 membrane trafficking, although the cytosolic cAMP concentration remains unaffected (Olesen et al., 2011). This might result from promiscuous G protein coupling (Fujino and Regan, 2006) which may occur upon PKA phosphorylation of EP_4_ (Olesen and Fenton, 2013). In line, EP_4_ activation stimulates phosphatidylinositol 3-kinase (PI3K) (Fujino et al., 2003), which is suggested to regulate AQP2 expression and endosomal retrieval (Tajika et al., 2004; Bustamante et al., 2005). Altogether, the molecular details of PGE_2_ signaling remain to be completely understood.

Non-steroidal anti-inflammatory drugs (NSAIDs) block the production of PGE_2_, other prostaglandins and thromboxanes by inhibition of the cyclooxygenases, COX-1 and COX-2 (Jin, 2015). NSAIDs are commonly used in the treatment of pain and inflammation. Their use in cell culture and animal models has shed some light on the role of prostaglandins such as PGE_2_ in the control of water homeostasis (Baggaley et al., 2010; Jia et al., 2012; Kortenoeven et al., 2012a).

Recently, effects of the chronic use of the NSAIDs, ibuprofen and meloxicam, on AQP2 were investigated in kidneys from Sprague-Dawley rats. The daily application of the drugs enhanced urine output and significantly reduced urine osmolality after 7–14 days. In line, both drugs significantly reduced the AQP2 protein abundance by 60–70%. Meloxicam significantly increased AQP2 phosphorylated at S256 and S261, whereas ibuprofen increased the AQP2 phosphorylated at S256 but did not affect the phosphorylation of AQP2 at S261. Both ibuprofen and meloxicam increased the phosphorylation of AQP2 at S264 and S269. The observed alterations in the C-terminal phosphorylations of AQP2 may partially compensate for the decrease in AQP2 abundance by promoting its membrane localization and thereby permitting only a limited effect on urine osmolality (Ren et al., 2015).

Indomethacin enhances the antidiuretic activity of AVP and markedly reduces urine output by increasing the AQP2 plasma membrane insertion. This effect of indomethacin is additive with that of thiazide diuretics (Kim et al., 2004b).

In a study where male Wistar rats were treated with indomethacin, ibuprofen and meloxicam AQP2 expression was significantly decreased in water-restricted rats but PGE_2_ excretion did not significantly change. This effect was less pronounced in water-loaded rats and meloxicam had no significant effect. The stronger effect of NSAIDs on the AQP2 protein level during water restriction seems most relevant for elderly and severely ill patients who are prone to dehydration (Baggaley et al., 2010; Lauridsen et al., 2010).

Stimulation of EP_2_ and EP_4_ increases AQP2 trafficking *via* increased cAMP. The EP_2_ agonist, butaprost relieved symptoms of NDI in a rat model (Gao et al., 2015; Olesen et al., 2011). Pharmacological blocking of V2R in rats results in NDI, which could be greatly mitigated *via* the administration of an EP_2_ agonist. EP_2_ agonists are already used in the treatment of dysmenorrhea (Moeller et al., 2013). Moreover, selective EP_4_ agonists relieved NDI symptoms developed in V2R gene-deficient mice (Bockenhauer and Bichet, 2014). In a mouse model for X-linked NDI, an EP_4_ receptor agonist, [ONO-AE-329 (ONO)] efficiently increased urine osmolality and reduced polyuria.

Taken together a possible strategy for the treatment of NDI might be the stimulation of EP_2_ and/or EP_4_ and simultaneous blocking of EP_3_.

PGE_2_ is particularly important for lithium-induced diabetes insipidus. Increased expression of COX-2 and abnormally high urinary excretion of PGE_2_ has been noted in animal models suffering from lithium-induced NDI. Lithium-treated patients have elevated urine PGE_2_ excretion as well (Kim et al., 2008). Administration of COX-2 inhibitors to adult mice and rats relieved the lithium-associated polyuria. Indomethacin blocks prostaglandin production in lithium-treated mpkCCD cells. Additionally, PGE_2_ synthase-1 (mPGES-1)-deficient mice did not develop lithium-associated polyuria (Jia et al., 2009; Baggaley et al., 2010; Kjaersgaard et al., 2014). In clinical practice inhibition of prostaglandin synthesis with NSAIDs remains an effective therapy for lithium-induced NDI (Kortenoeven et al., 2012a).

### PDE inhibitors

An increase in cGMP levels, e.g., by ANP or the nitric oxide (NO) donor sodium nitroprusside activates protein kinase G (PKG). PKG can phosphorylate AQP2 at S256 to induce its redistribution into the plasma membrane (Bouley et al., 2000). PDE5 specifically hydrolyses cGMP and thereby prevents cGMP/PKG signaling. PDE5 is present in the principal cells of the collecting duct along with further PDEs, including PDE1 and PDE4 (Dousa, 1999). Inhibition of PDE5, in particular with sildenafil (Viagra) causes AQP2 plasma membrane accumulation in LLC-PK_1_ cells. This effect was attributed to the activation of the NO/cGMP pathway (Bouley et al., 2000, 2005) as also sodium nitroprusside, L-arginine (a precursor of NO) and ANP induce plasma membrane accumulation of AQP2 *in vitro*, and ANP also *in vivo* (Brown et al., 2012). In line, mice deficient in NO synthase isoforms developed NDI (Morishita et al., 2005a). PDE5 inhibitors and ANP combined cause natriuresis and decreased urine output (Bouley et al., 2005; Assadi and Ghane Sharbaf, 2015). Therefore, the activation of AVP-independent cGMP signaling with sildenafil could be an option in the treatment of X-linked NDI as it bypasses the defective V2R/cAMP axis (Sanches et al., 2012). Sildenafil is already used for the treatment of erectile dysfunction, pulmonary hypertension and subarachnoid hemorrhage.

The PDE4 family of enzymes (PDE4A, PDE4B, PDE4C, and PDE4D) encodes more than 20 isozymes (Houslay, 2010; Klussmann, 2015). Rolipram selectively inhibits the PDE4 isozymes by targeting the catalytic domain of the enzymes. Rolipram enhances the AVP-induced accumulation of cAMP, the translocation of AQP2 into the plasma membrane and the osmotic water permeability of cultured IMCD cells (Stefan et al., 2007). Moreover, rolipram increased urine osmolality in a mouse model of autosomal dominant NDI (Sohara et al., 2006b). However, the clinical trial with rolipram failed (Bichet et al., 1990) because of its intolerable side effects, mainly nausea and vomiting. Recently, roflumilast, and apremilast, both non-selective inhibitors of PDE4A-D, with fewer side effects reached the market. Roflumilast is approved for the treatment of chronic obstructive pulmonary disease (COPD) and apremilast for psoriatic arthritis (Klussmann, 2015). These new PDE4 inhibitors may provide a new opportunity for the therapy of X-linked NDI.

### Pharmacological chaperones

Cell-permeable pharmacological chaperons have proven their efficacy in restoring the plasma membrane localization and functional rescue of V2R. Vaptans (see below) not only block the V2R but can also act as such chaperones. The cell-permeable V2R antagonists S121463 and VPA-985 stabilize ER-retained V2R mutants, so that the receptor can be released from the ER and inserted into the plasma membrane (Morello et al., 2000; Wüller et al., 2004). These positive effects of pharmacological chaperons were reproducible in Madin-Darby canine kidney (MDCK) cells overexpressing nine ER-retained V2R mutants involved in NDI (Robben et al., 2006). SR49059 (Conivaptan), a non-selective blocker of V2R and vasopressin V1a receptors (Bernier et al., 2004), has been tested in NDI patients where it decreased urine output and was thus suggested for the treatment of X-linked NDI (Bernier et al., 2006). The chaperones are active in nanomolar concentrations on cultured cells. Regarding NDI treatment the high affinity OPC31260 (mozavaptan) and OPC41061 (tolvaptan) non-peptidic antagonists would promise beneficial effects in terms of V2R rescue, low doses and efficient substitution for AVP (Robben et al., 2007; Wesche et al., 2012). On the other hand, since low affinity antagonists require higher doses for clinical efficacy this could lead to hepatic toxicity because of potential interference with cytochrome P450 systems. Hence, further studies using modified antagonists or treatment regimes are required.

*Vice versa*, agonists binding to V2R mutants induced the production of sufficient amounts of cAMP and the translocation of AQP2 into the apical plasma membrane of renal cells. Biased agonists of the V2R represent a novel therapeutic strategy for the treatment of congenital NDI. The non-peptidic agonists MCF14 (OPC23h), MCF18 (VNA932), and MCF57 are full agonist of the V2R but do not induce receptor internalization and arrestin-associated signaling. From a clinical perspective these properties can be of great importance as they can have long-lasting effects (Mouillac and Mendre, 2014).

Both strategies with antagonists or agonists are not applicable if V2R is not at least partially functional or if it is insensitive to such agents (Moeller et al., 2013; Bockenhauer and Bichet, 2014).

### Activators of the AQP2 shuttle

An unbiased screening of 3646 compounds for stimulators of the redistribution of AQP2 to the apical plasma membrane of LLC-PK_1_ cells identified the JAK-2 and EGFR inhibitor AG-490. The molecule induced the AQP2 accumulation at the plasma membranes of LLC-PK_1_ and Madin-Darby canine kidney (MDCK) cells and at that of principal cells in rat kidney sections. AG-490 treatment of AVP-deficient Brattleboro rats improved their urine concentrating capacity within 2 h (Nomura et al., 2014; Sands and Blount, 2014).

## Pharmacological treatments of water balance disorders with enhanced AVP-mediated water reabsorption and novel avenues for therapy

### Vaptans

Vaptans, also termed aquaretics, comprise a family of V2R antagonists. They include intravenously administered conivaptan and oral vaptans like tolvaptan, lixivaptan, and satavaptan (Narayen and Mandal, 2012). Tolvaptan, the most extensively used vaptan, selectively inhibits the binding of AVP to the V2R with an almost two times higher affinity than AVP, and interferes with the AQP2 redistribution into the plasma membrane of collecting duct principal cells (Sato et al., 2014). Patients treated with tolvaptan have increased urine AQP2 levels (Sato et al., 2014), indicative of shedding of AQP2 from the apical plasma membrane into the urine.

The aquaretic effect is achieved by enhanced free water clearance without induction of natriuresis, kaliuresis or changes of the glomerular filtration rate (Graziani et al., 2014). Vaptans are approved for the therapy of hypervolemic (heart failure, liver cirrhosis, SIADH) (Narayen and Mandal, 2012) and normovolemic hyponatremia, and since recently for autosomal dominant polycystic kidney disease (Lin et al., 2014; Blair and Keating, 2015). In particular, the adjustment of sodium levels in patients suffering from SIADH and hyponatremia can be easier controlled with vaptans than with conventional hypertonic saline infusions. This translates into a better safety profile, and a lower risk of side effects (Nathan, 2007). Heart failure patients are treated with conventional anti-hypertensive drugs and only since recently additionally with V2 receptor antagonists (vaptans, see below) if hospitalized (Gilotra and Russell, 2014). More than half of the cirrhosis patients suffer from ascites, a major complication associated with sodium and AVP-dependent water retention that is characterized by the accumulation of fluid in the abdominal cavity. Hence, V2R antagonists could prove beneficial in the symptomatic treatment of this condition by increasing urine output (Yan et al., 2015).

### Demeclocycline

Demeclocycline, a bacteriostatic antibiotic of the tetracycline group, is used for the treatment of sustained hyponatremia in patients with SIADH. It is the first choice in this case because of its aquaretic effect. Demeclocycline decreases the AVP-induced cAMP production and abundance of adenylyl cyclases 3 and 5/6, and reduces the expression of AQP2 in the apical plasma membrane of mpkCCD cells without affecting the V2R. In a rat model of SIADH, demeclocycline increased urine volume, decreased urine osmolality and alleviated hyponatremia as has been shown in patients (Kortenoeven et al., 2013). Other tetracycline antibiotics including minocycline, doxycycline and tetracycline have similar effects with regard to reducing urine volume in toads (Feldman and Singer, 1974; Hirji and Mucklow, 1991). Tetracycline reduces urinary concentrating ability also in humans (Wilson et al., 1973). However, demeclocycline (600–1200 mg/day) may also cause NDI in some patients, and that is the reason for the limited application of these drugs in clinical practice (Gross, 2012).

## Pharmacological targeting of protein-protein interactions for interference with AQP2 trafficking

Protein-protein interactions play key roles in all biological processes. They are diverse and highly specific and, therefore, provide ample opportunities for the development of novel drugs (Jones and Thornton, 1996; Klussmann and Rosenthal, 2008; Cho et al., 2016). Since AKAP-PKA interactions are involved in AQP2 trafficking (Klussmann et al., 1999; Klussmann and Rosenthal, 2001), their disruption may lead to a new concept for the treatment of diseases characterized by excessive AVP-mediated water retention such as heart failure (Nedvetsky et al., 2009; Tröger et al., 2012; Deak and Klussmann, 2015; Dema et al., 2015). Indeed, peptides, peptidomimetics, and small molecules for the non-selective disruption of AKAP-PKA interactions are available (Hundsrucker et al., 2006a,b; Hundsrucker and Klussmann, 2008; Christian et al., 2011; Schäfer et al., 2013; Schächterle et al., 2015). All of these agents target the AKAP-binding domain of regulatory R subunits of PKA, the dimerization/docking (D/D) domain. They bind competitively and thereby prevent the interactions of R subunits with AKAPs (Deak and Klussmann, 2015). The peptides such as Ht31, AKAP18-derived peptides or AKAP_IS_ all bind R subunits with high affinity and thereby effectively disrupt AKAP-PKA interactions. However, short half-life, low bioavailability, and membrane permeability limit the development of peptide-based drugs, although several peptide-drugs reached the market (Otvos and Wade, 2014). The available peptidomimetics are low affinity binders and require a multistep synthesis. They would require further optimization prior to becoming candidates for drug development (Dema et al., 2015). Most promising are small molecule inhibitors of protein-protein interactions. With good bioavailability and enhanced plasma membrane permeability they can be optimized toward effective inhibitors. However, the development is challenging, because protein-protein interaction interfaces are generally large and shallow and can hardly be completely covered by small molecules. Despite this, in the past years remarkable progress has been made and several small molecule inhibitors of protein-protein interactions were discovered (Sheng et al., 2015). The only small molecule inhibiting AKAP-PKA interactions, FMP-API-1, is not suitable for further development toward a drug candidate as it also activates PKA (Christian et al., 2011; Yu et al., 2014).

In order to identify small molecule inhibitors of the AVP-induced redistribution of AQP2, high-throughput screening of small molecule libraries provides ample opportunities. A prototypical example is a screening of 17,700 small molecules for inhibitors of the cAMP-induced redistribution of AQP2 using a cell-based assay (Bogum et al., 2013). This approach yielded 17 inhibitors, among them, 4-acetyldiphyllin (4-AD) a selective blocker of V-ATPase. 4AD increased pH levels of intracellular vesicles and caused an accumulation of AQP2 in the Golgi. The compound prevented the PKA-induced phosphorylation of AQP2 at S256 without affecting cAMP levels, PKA activity or the phosphorylations of AQP2 at S261 and S269. Although the screening approach did not yield an inhibitor of PPIs, it is suitable for identification of small molecules that may serve as a starting point for the development of novel drug candidates for the therapy of disorders associated with increased AQP2 levels (Bogum et al., 2013).

## Summary and outlook

The localization of AQP2 is critical for the reabsorption of water. If located in the plasma membrane of renal collecting duct principal cells AQP2 facilitates water reabsorption from primary urine. A predominant localization in the plasma membrane contributes to excessive water retention as in heart failure, SIADH or liver cirrhosis; *vice versa* a predominant intracellular localization causes diabetes insipidus, a disease characterized by a massive loss of hypotonic urine. A variety of cAMP-dependent and independent signaling pathways and defined protein-protein interactions have been identified that control the localization of AQP2. Considering the still unmet medical need in both classes of diseases, i.e., diseases with a high- or a low-level plasma membrane localization of AQP2, pharmacological targeting of the mechanisms controlling AQP2 trafficking provides ample opportunities for the development of novel drugs.

Several established drugs modulate the localization of AQP2 in animal models, and first data from patients confirmed these effects. Examples are statins that promote or NSAIDs and vaptans that prevent the plasma membrane localization of AQP2. Further clinical trials will have to confirm the value of repurposing such drugs toward the treatment of water balance disorders. However, all of these established drugs cause unwanted side effects, which may limit their use. Therefore, it will be necessary to continue with the identification of molecular mechanisms underlying the control of the localization of AQP2 to provide novel drug targets. In particular, identifying protein-protein interactions that govern the control of AQP2 is relevant as protein-protein interactions are highly specific and diverse and therefore represent a unique class of drug targets. AKAP-PKA interactions that are essential for the AVP-induced redistribution of AQP2 are an example (Christian et al., 2011; Schächterle et al., 2015). It is important to define “hot spots” within the interacting surfaces, i.e., crucial amino acids that mediate the interaction and that can be targeted with small molecules for effective disruption (Buchwald, 2010; Villoutreix et al., 2014; Sheng et al., 2015). For identifying small molecule inhibitors of protein-protein interactions high-throughput screening may be used. This approach has led to the identification of FMP-API-1, the first small molecule disruptor of AKAP-PKA interactions (Keskin et al., 2008; Christian et al., 2011).

Other unbiased screening approaches have identified small molecule modulators of the AVP-induced redistribution of AQP2. The identification of the inhibitor 4AD for example provided new insight into the regulation of AQP2. It identified V-ATPase as a protein involved in the control of AQP2 (Bogum et al., 2013). Another example is the identification of a small molecule activator of the AQP2 redistribution, AG-490. AG-490 inhibits the EGF receptor and JAK-2 kinase (Nomura et al., 2014). Even though targeting a ubiquitous protein such as V-ATPase or the EGF receptor may not be an option for the treatment of patients, the screening approaches are valuable for the identification of new molecules and targets and may provide new starting points for the development of novel drugs for the treatment of water balance disorders.

Taken together, many questions surrounding water balance disorders have been answered but even more remain unanswered. A particular topic in the future will be to translate the insights into molecular mechanisms underlying the trafficking of AQP2 into novel therapies for these diseases with their unmet medical needs.

## Author contributions

TV and MS contributed equally. TV wrote most of the pharmacology section, MS most of the section relating to molecular mechanisms underlying AQP2 trafficking and the figures. DF contributed the tables and to sections about AQP2 trafficking. EK designed the study wrote parts of all sections and edited the manuscript.

## Funding

This work was supported by grants from the Else Kröner-Fresenius-Stiftung (2013_A145), the German-Israeli Foundation (G.I.F. I-1210-286.13/2012), and the German Centre for Cardiovascular Research (DZHK 81X210012) to EK.

### Conflict of interest statement

The authors declare that the research was conducted in the absence of any commercial or financial relationships that could be construed as a potential conflict of interest.
